# Supplementary material for: Effectiveness and adequacy of blinding in the moderation of pain outcomes: Systematic review and meta-analyses of dry needling trials
Source: PeerJ. 2018 Jul 31;6:e5318. doi: 10.7717/peerj.5318 (PMC6074757; doi:10.7717/peerj.5318)
Supplement: Supplemental Information 1 [file peerj-06-5318-s001.docx]

Note: Further details including text excerpts from the manuscript to explain how each PRISMA item was accomplished are presented after the table.

PRISMA Table

|  | **#** | **Checklist item** | **Reported on page #** |
| --- | --- | --- | --- |
| **TITLE** | | |  |
| Title | 1 | Identify the report as a systematic review, meta-analysis, or both. | 1 |
| **ABSTRACT** | | |  |
| Structured summary | 2 | Provide a structured summary including, as applicable: background; objectives; data sources; study eligibility criteria, participants, and interventions; study appraisal and synthesis methods; results; limitations; conclusions and implications of key findings; systematic review registration number. | 2-3 |
| **INTRODUCTION** | | |  |
| Rationale | 3 | Describe the rationale for the review in the context of what is already known. | 4-5 |
| Objectives | 4 | Provide an explicit statement of questions being addressed with reference to participants, interventions, comparisons, outcomes, and study design (PICOS). | 5 |
| **METHODS** | | |  |
| Protocol and registration | 5 | Indicate if a review protocol exists, if and where it can be accessed (e.g., Web address), and, if available, provide registration information including registration number. | 6 |
| Eligibility criteria | 6 | Specify study characteristics (e.g., PICOS, length of follow-up) and report characteristics (e.g., years considered, language, publication status) used as criteria for eligibility, giving rationale. | 6-7 |
| Information sources | 7 | Describe all information sources (e.g., databases with dates of coverage, contact with study authors to identify additional studies) in the search and date last searched. | 6 |
| Search | 8 | Present full electronic search strategy for at least one database, including any limits used, such that it could be repeated. | Table 1  6 |
| Study selection | 9 | State the process for selecting studies (i.e., screening, eligibility, included in systematic review, and, if applicable, included in the meta-analysis). | 7 |
| Data collection process | 10 | Describe method of data extraction from reports (e.g., piloted forms, independently, in duplicate) and any processes for obtaining and confirming data from investigators. | 7-8 and 11 |
| Data items | 11 | List and define all variables for which data were sought (e.g., PICOS, funding sources) and any assumptions and simplifications made. | 7-8 |
| Risk of bias in individual studies | 12 | Describe methods used for assessing risk of bias of individual studies (including specification of whether this was done at the study or outcome level), and how this information is to be used in any data synthesis. | 8 |
| Summary measures | 13 | State the principal summary measures (e.g., risk ratio, difference in means). | 9 |
| Synthesis of results | 14 | Describe the methods of handling data and combining results of studies, if done, including measures of consistency (e.g., I^2^) for each meta-analysis. | 8-10 |
| Risk of bias across studies | 15 | Specify any assessment of risk of bias that may affect the cumulative evidence (e.g., publication bias, selective reporting within studies). | 8 |
| Additional analyses | 16 | Describe methods of additional analyses (e.g., sensitivity or subgroup analyses, meta-regression), if done, indicating which were pre-specified. | 8-10 |
| **RESULTS** | | |  |
| Study selection | 17 | Give numbers of studies screened, assessed for eligibility, and included in the review, with reasons for exclusions at each stage, ideally with a flow diagram. | 10-11  Fig. 1 |
| Study characteristics | 18 | For each study, present characteristics for which data were extracted (e.g., study size, PICOS, follow-up period) and provide the citations. | Table 4 |
| Risk of bias within studies | 19 | Present data on risk of bias of each study and, if available, any outcome level assessment (see item 12). | 11  Table 3 |
| Results of individual studies | 20 | For all outcomes considered (benefits or harms), present, for each study: (a) simple summary data for each intervention group (b) effect estimates and confidence intervals, ideally with a forest plot. | 12-14  Table 4  Fig. 3-6 |
| Synthesis of results | 21 | Present results of each meta-analysis done, including confidence intervals and measures of consistency. | 12-14  Fig. 3-6 |
| Risk of bias across studies | 22 | Present results of any assessment of risk of bias across studies (see Item 15). | 11  Fig. 2 |
| Additional analysis | 23 | Give results of additional analyses, if done (e.g., sensitivity or subgroup analyses, meta-regression [see Item 16]). | 12-14  Fig. 3-6 |
| **DISCUSSION** | | |  |
| Summary of evidence | 24 | Summarize the main findings including the strength of evidence for each main outcome; consider their relevance to key groups (e.g., healthcare providers, users, and policy makers). | 14-18 |
| Limitations | 25 | Discuss limitations at study and outcome level (e.g., risk of bias), and at review-level (e.g., incomplete retrieval of identified research, reporting bias). | 18-19 |
| Conclusions | 26 | Provide a general interpretation of the results in the context of other evidence, and implications for future research. | 19-20 |
| **FUNDING** | | |  |
| Funding | 27 | Describe sources of funding for the systematic review and other support (e.g., supply of data); role of funders for the systematic review. | 20 |

*From:*  Moher D, Liberati A, Tetzlaff J, Altman DG, The PRISMA Group (2009). Preferred Reporting Items for Systematic Reviews and Meta-Analyses: The PRISMA Statement. PLoS Med 6(7): e1000097. doi:10.1371/journal.pmed1000097

For more information, visit: **www.prisma-statement.org**.

**TITLE**

**Item 1: Title - Identify the report as a systematic review, meta-analysis, or both.**

Page 1: Adequacy and effectiveness of blinding in the moderation of pain outcomes: systematic review and meta-analyses of dry needling trials

**ABSTRACT**

**Item 2: Structured summary - Provide a structured summary including, as applicable: background; objectives; data sources; study eligibility criteria, participants, and interventions; study appraisal and synthesis methods; results; limitations; conclusions and implications of key findings; systematic review registration number.**

Page 2-3: Abstract

**INTRODUCTION**

**Item 3: Rationale - Describe the rationale for the review in the context of what is already known.**

Page 4-5: Background

**Item 4: Objectives - Provide an explicit statement of questions being addressed with reference to participants, interventions, comparisons, outcomes, and study design (PICOS).**

Page 5: the aim of this systematic review was to determine the influence of blinding effectiveness and blinding adequacy on pain in sham-controlled dry needling trials. Blinding effectiveness was determined by participant beliefs about group allocation relative to actual allocation, and blinding adequacy was determined by critical appraisal. There were two questions: (1) ‘Does blinding effectiveness moderate intervention effect on pain?’ and (2) ‘Does blinding adequacy moderate intervention effect on pain?’.

Note: all prospective experimental designs were eligible and there was no limit on participant health condition (p. 6).

**METHODS**

**Item 5: Protocol and registration - Indicate if a review protocol exists, if and where it can be accessed (e.g., Web address), and, if available, provide registration information including registration number**.

Page 6: The protocol was prospectively registered with the International Prospective Register of Systematic Reviews (PROSPERO) (registration number: 42016029340; URL: [www.crd.york.ac.uk/prospero/display_record.asp?ID=CRD42016029340](http://www.crd.york.ac.uk/prospero/display_record.asp?ID=CRD42016029340)).

**Item 6: Eligibility criteria - Specify study characteristics (e.g., PICOS, length of follow-up) and report characteristics (e.g., years considered, language, publication status) used as criteria for eligibility, giving rationale.**

Page 6-7: Trials were eligible for inclusion in this review if they 1) were prospective experimental designs (e.g. randomised, non/quasi-randomised trials, pre-post, n-of-1) of any duration, which included a ‘real’ dry needling intervention (referred to as ‘active’ dry needling in this review) and a placebo/sham dry needling intervention; 2) included human adults (>18 years of age) who were asymptomatic or with symptomatic health conditions; 3) involved a recognised dry needling approach with needle insertion sites based on anatomical or clinical rationales; 4) assessed and reported an outcome for pain [visual analogue scale (VAS) or numeric rating scale (NRS)]. Trials were also eligible for inclusion if they reported blinding assessment data, without reporting on pain, but the results from these trials are not presented in this review. Trials were ineligible for inclusion if the needling therapy involved pre-designated needle insertion sites (e.g. traditional acupuncture points) or involved injection of a substance (wet needling).

Page 6: There were no limits on year, language, or publication status.

**Item 7: Information sources - Describe all information sources (e.g., databases with dates of coverage, contact with study authors to identify additional studies) in the search and date last searched.**

Page 6: Databases (MEDLINE, EMBASE, AMED, Scopus, CINAHL, PEDro, The Cochrane Library) were searched from inception to February 2016. Thesis databases (Trove, ProQuest) and clinical trial registries [Australian New Zealand Clinical Trials Registry (ANZCTR), Clinicaltrials.gov, World Health Organization International Clinical Trials Registry Platform (WHO ICTRP)] were crosschecked with database searches to identify further potential studies. The reference lists of systematic reviews identified by the search were examined to locate additional or unpublished studies. There were no limits on year, language, or publication status.

**Item 8: Search - Present full electronic search strategy for at least one database, including any limits used, such that it could be repeated.**

Table 1: Presents the search strategy used in MEDLINE.

Page 6: The general search terms were (needl* OR acupuncture OR intramuscular stimulation) AND (sham OR placebo*), and Medical Subject Headings (MeSH) were used where possible. Searches were modified to suit the functionality of each database.

**Item 9: Study selection - State the process for selecting studies (i.e., screening, eligibility, included in systematic review, and, if applicable, included in the meta-analysis).**

Page 7: Records identified from the search strategy were exported to Endnote, duplicates were removed, and the remaining records were imported into the screening and data extraction tool ‘Covidence systematic review software’ (2018). Titles and abstracts were screened against the eligibility criteria by three independent reviewers in teams of two (FAB and MPM or JLW), and studies potentially meeting the criteria were progressed to full text review. The same three reviewers independently screened the full-text articles in teams of two. Discrepancies were resolved through discussion, with an independent third reviewer (MPM, JLW, or LSKL) consulted where necessary. Where full-text was unavailable, authors were contacted to clarify eligibility and/or to provide full-texts.

**Item 10: Data collection process - Describe method of data extraction from reports (e.g., piloted forms, independently, in duplicate) and any processes for obtaining and confirming data from investigators.**

Page 7: A prospectively designed data extraction template was developed based on the Standards for Reporting Interventions in Controlled Trials of Acupuncture (STRICTA) (MacPherson et al. 2010) and the Cochrane Handbook ‘Checklist of items to consider in data collection or data extraction’ (Higgins & Green 2011).

Page 8: The provisional data extraction template was pilot tested for inter-rater agreement by two reviewers (FAB and JLW) using an iterative process (two randomly selected included studies in each iteration). Once the pre-specified level of inter-rater agreement was established (>75% agreement of items within an individual trial), two independent reviewers performed the remaining data extraction (FAB and LSKL, JLW, or MPM), with a third reviewer consulted to resolve disagreements as required.

Page 11: Nine authors of included trials were contacted to clarify trial details or request data; one author replied, stating they no longer had access to the data, and the remaining eight authors did not reply.

**Item 11: Data items - List and define all variables for which data were sought (e.g., PICOS, funding sources) and any assumptions and simplifications made.**

Page 7: The domains of data extraction were: source details, study demographics, study design, participant details, therapist details, intervention details, outcomes (pain and blinding assessment), blinding strategies, sample size and dropouts, results (pain and blinding assessment), and key conclusions of the authors.

Page 8: Only data from the first phase of crossover trials were extracted due to the risk of carry-over intervention effects. Where necessary (i.e. where no text or table data provided), graphical data was extracted using a ruler; if there were differences in these values between the two extracting reviewers, the average value was calculated. Pain intensity data were converted to a 100-point scale where required (e.g. if collected using a 10cm VAS or an NRS).

**Item 12: Risk of bias in individual studies - Describe methods used for assessing risk of bias of individual studies (including specification of whether this was done at the study or outcome level), and how this information is to be used in any data synthesis.**

Page 8: Risk of Bias (RoB) of individual trials was assessed using the Cochrane RoB assessment tool for randomised trials (as all included studies were randomised trials) (Higgins et al. 2011). Three key domains (allocation concealment, performance bias, detection bias) were determined *a priori* based on relevance to the review questions. An additional domain (chance) was added to the tool (under ‘other biases’), for which RoB assessments were based on the achievement of sufficient power for pain outcomes. The key domains and the additional domain were informed by empirical evidence for the likelihood and magnitude of these biases influencing trial outcomes (Button et al. 2013; Higgins et al. 2011; Hróbjartsson et al. 2014; Hróbjartsson et al. 2013; Savović et al. 2012; Wood et al. 2008). The overall RoB for individual trials was determined using the three key domains (low = low RoB for all key domains, unclear = low or unclear RoB for all key domains, high = high RoB for one or more key domains) (Higgins et al. 2011). Two independent reviewers appraised each trial (FAB and MPM, JLW, or LSKL), with a third reviewer consulted to resolve disagreements as required.

**Item 13: Summary measures - State the principal summary measures (e.g., risk ratio, difference in means).**

Page 9: Difference in means of active versus sham intervention effects (pain) [Standardised Mean Difference (SMD)], blinding Index (BI) (Bang et al. 2004).

**Item 14: Synthesis of results - Describe the methods of handling data and combining results of studies, if done, including measures of consistency (e.g., I^2^) for each meta-analysis.**

Page 8-10: Meta-regression was used for review question 1, subgroup meta-analyses were used for review question 2; further details of the data synthesis methods provided on pages 8-10.

Page 8: For both review questions, meta-analyses used generic inverse variance and random-effects models. Stata statistical software (version 15.1) (StataCorp. 2017) was used to compute inferential statistics and create plots. The x^2^ test and *I^2^* statistic were used to assess statistical heterogeneity; p<0.10 was interpreted as statistically significant heterogeneity and *I^2^* >50% was interpreted as substantial heterogeneity (Higgins & Green 2011).

**Item 15: Risk of bias across studies - Specify any assessment of risk of bias that may affect the cumulative evidence (e.g., publication bias, selective reporting within studies).**

Page 8: Publication bias for each meta-analysis was assessed by visual inspection of asymmetry of funnel plots, which were contour-enhanced to allow consideration of the potential influence of the statistical significance of trial outcomes on publication bias (Peters et al. 2008). A statistical test for asymmetry was also computed for funnel plots containing ≥10 trials using the method specified in Egger et al. (1997) at a significance level of p<0.10 (Higgins & Green 2011; Sterne et al. 2011).

**Item 16: Additional analyses - Describe methods of additional analyses (e.g., sensitivity or subgroup analyses, meta-regression), if done, indicating which were pre-specified.**

Page 8-10: Meta-regression was used for review question 1, subgroup meta-analyses were used for review question 2. All analyses were pre-specified.

**RESULTS**

**Item 17: Study selection - Give numbers of studies screened, assessed for eligibility, and included in the review, with reasons for exclusions at each stage, ideally with a flow diagram.**

Fig. 1: PRISMA flow diagram

Page 10-11: The outcome of the search strategy is presented in Fig. 1. The search identified 11835 records. Four additional publications were identified by searching personal records (Itoh et al. 2004) and through hand searching reference lists of 199 systematic reviews (Itoh & Katsumi 2005; Itoh et al. 2006; Katsumi et al. 2004). Following removal of duplicates, 4894 potentially relevant publications were screened. Title and abstract screening excluded 4280 publications. Of the remaining 614 publications, 588 were excluded following full-text review, leaving 26 publications (Fig. 1). The exclusion of two research questions from this review resulted in the exclusion of three trials (within one thesis) from the current review as they did not report a pain outcome (Braithwaite 2014) (this publication is included in Fig. 1 because the two omitted review questions that did include results from this publication are reported elsewhere). Accounting for one trial that presented results over two publications (Tough et al. 2010; Tough et al. 2009), and two single publications with two eligible sham groups (Itoh & Katsumi 2005; Itoh et al. 2007), 25 publications (with 26 group comparisons from 24 trials) are presented in the current review. Of these 25 publications, 24 publications (with 25 group comparisons from 23 trials) provided sufficient data for inclusion in the meta-analyses (Fig. 1). For the meta-analyses, in the two trials with two eligible sham groups (Itoh & Katsumi 2005; Itoh et al. 2007) the active group data were used twice.

**Item 18: Study characteristics - For each study, present characteristics for which data were extracted (e.g., study size, PICOS, follow-up period) and provide the citations.**

Table 4: Characteristics and results of included group comparisons

**Item 19: Risk of bias within studies - Present data on risk of bias of each study and, if available, any outcome level assessment (see item 12).**

Table 3: Risk of bias assessment

Page 11: A summary of results for the RoB assessment is presented in Table 3. Overall RoB was high in one trial, unclear in 18 trials and low in five trials (Table 3). The areas with least RoB were participant blinding and reporting bias (low RoB in all included trials). The areas with greatest potential for bias were blinding of therapists and research personnel (high or unclear RoB in all included trials), allocation concealment, and attrition bias (Table 3).

**Item 20: Results of individual studies - For all outcomes considered (benefits or harms), present, for each study: (a) simple summary data for each intervention group (b) effect estimates and confidence intervals, ideally with a forest plot.**

Table 4: Characteristics and results of included group comparisons

Fig. 3 (bubble plot of meta-regression); Fig. 4-6 (forest plots)

Results also presented in text on pages 12-14

**Item 21: Synthesis of results - Present results of each meta-analysis done, including confidence intervals and measures of consistency.**

Fig. 3 (plot of meta-regression); Fig. 4-6 (forest plots)

Results also presented in text on pages 12-14

**Item 22: Risk of bias across studies - Present results of any assessment of risk of bias across studies (see Item 15).**

Fig. 2: Contour-enhanced funnel plots

Page 11: Visual inspection of asymmetry of contour-enhanced funnel plots suggested that publication bias may be present (Fig. 2) (Peters et al. 2008). A statistical test for asymmetry was computed for funnel plots containing ≥10 group comparisons (Fig. 2A, 2C, and 2D) and a significant result was found for all three plots (p=0.00, p=0.083, and p=0.061 respectively), which further supports the presence of publication bias (Egger et al. 1997).

**Item 23: Additional analyses - Give results of additional analyses, if done (e.g., sensitivity or subgroup analyses, meta-regression [see Item 16]).**

See items 20 and 21

**DISCUSSION**

**Item 24: Summary of evidence - Summarize the main findings including the strength of evidence for each main outcome; consider their relevance to key groups (e.g., healthcare providers, users, and policy makers).**

Page 14-15 Discussion for review question 1

Page 15-18 Discussion for review question 2

**Item 25: Limitations - Discuss limitations at study and outcome level (e.g., risk of bias), and at review-level (e.g., incomplete retrieval of identified research, reporting bias).**

Page 18-19: Limitations are discussed in detail

**Item 26: Conclusions - Provide a general interpretation of the results in the context of other evidence, and implications for future research.**

Page 19-20: Conclusions

**Item 27: Funding - Describe sources of funding for the systematic review and other support (e.g., supply of data); role of funders for the systematic review.**

Page 20: Conflicts of interest statements and research funding: FAB and LSKL were each supported by an Australian Government Research Training Program Scholarship. JLW, MTW, and MPM had no financial or other relationships that might lead to a conflict of interest (real or perceived). GLM has received support from Pfizer, Kaiser Permanente, Providence Healthcare, Agile Physiotherapy, Results Physiotherapy, Workers' Compensation Boards in Australia, Europe and North America, the International Olympic Committee and the Port Adelaide Football Club. GLM receives royalties for several books on pain and speaker’s fees for talks on pain, physiotherapy, and rehabilitation. GLM was supported by a Principal Research Fellowship from the National Health and Medical Research Council of Australia (NHMRC) ID 1061279.
